# The Informal, Non-Prescribed Use of Antiretroviral Medications for PrEP Among a National US-Based Sample of Gay, Bisexual, and Other Men Who Have Sex with Men: A Cross-Sectional Study

**DOI:** 10.1007/s10461-024-04589-4

**Published:** 2024-12-30

**Authors:** Mance E. Buttram, Matthew S. Ellis, Krishen D. Samuel, Matthew Hayhurst

**Affiliations:** 1 Department of Health, Human Performance, and Recreation, University of Arkansas, Fayetteville, AR USA; 2 Center for Public Health and Technology, University of Arkansas, Fayetteville, AR USA; 3 Department of Psychiatry, Washington University in St. Louis School of Medicine, St. Louis, MO USA

**Keywords:** PrEP, Informal PrEP, HIV prevention, Sexual behaviors, Men who have sex with men, GBM

## Abstract

This brief report presents findings on informal, non-prescribed PrEP use among an online sample of gay, bisexual and other men who have sex with men (*n* = 196). Mean age was 33.4. Participants were Hispanic (13.3%), African American (15.8%), white (63.8%), and other race/ethnicity (6.6%). Informal PrEP users (11%) more frequently reported past year sexually transmitted infections (*p* < 0.001), group sex (*p* < 0.001), sex in public (*p* < 0.01), transactional sex (*p* < 0.001), ever receiving a formal PrEP prescription (*p* < 0.05), and ease of finding diverted HIV medications on gay dating/sex apps (*p* < 0.05). Formal PrEP uptake should be encouraged to mitigate potential negative consequences of informal use.

## Introduction

Daily oral pre-exposure prophylaxis (PrEP) is a proven biomedical HIV prevention intervention [1]. As of 2021, it is estimated that of the 1.2 million individuals in the US who would likely benefit from PrEP, 30% have been prescribed, which is a substantial increase from 13% in 2017 [2]. The highest rates of increase in PrEP uptake are among gay, bisexual, and other men who have sex with men (GBM) [3].

At the same time, there are documented examples of non-prescribed, non-medically supervised use of HIV antiretroviral medications for HIV prevention that were obtained from a source other than a healthcare provider (hereafter “informal PrEP”) [4, 5]. Data on informal PrEP use among GBM in the US are limited to one apparent study and results show that men report a range of medications and dosing schemes that do not cohere with approved regimens [4]. Moreover, two participants in that same study reported HIV seroconversion after engaging in informal PrEP use [6].

There exist several complexities surrounding PrEP which may limit uptake of formal, prescribed PrEP, including approval of new medications and formulations, varying dosage schemes (e.g., daily vs. “on demand” or event driven), cost of medication and associated medical monitoring, and structural, interpersonal, and individual-level barriers, especially among non-white GBM [7, 8]. Thus, the aim of this brief report was to examine informal PrEP use among a US sample of GBM, with a focus on demographics, sexual behaviors, and PrEP experience, knowledge, and attitudes.

## Methods

### Sampling and Recruitment

Data are drawn from an anonymous cross-sectional online survey of GBM administered in June 2023 via Prolific, an online research recruitment platform. Eligibility criteria included being aged 18–55, identifying as LGBTQ, reporting sex with men in the past 6 months, residing in the US, and use of smartphone enabled dating applications. Informal PrEP use was defined for participants as obtaining HIV antiretroviral medication from a source other than a doctor, pharmacy, or healthcare provider and taking it for HIV prevention. All participants provided informed consent prior to completing the survey. In addition, participants received a stipend of $10 for participation. This study was approved by the university Institutional Review Board.

## Measures

Demographic measures included age, race/ethnicity, and education; participants reported past year diagnosis of a sexually transmitted infection (i.e., syphilis, gonorrhoea, or chlamydia). Residential ZIP codes were collected and categorized using the National Center for Health Statistics’ Urban-Rural classification scheme [9] and dichotomized for analysis into large central metropolitan county vs. not; large central metropolitan counties are in metropolitan statistical areas (MSAs) of at least one million population and contain the entire population of the principal city in the MSA, or are completely contained within the principal city of the MSA, or contain at least 250,000 residents of the principal city in the MSA. Using the Movement Advancement Project’s designations of state-level LGBTQ equality derived from local laws and policies [10], participant ZIP codes were also categorized into residing in a location with positive LGBTQ + policy vs. not.

Sexual behavior measures included past 6-month male and female sex partners, as well as past 12-month participation in group sex (i.e., three or more people at one time), sex in public (i.e., gay bathhouse or sauna), and transactional sex (i.e., using money, gifts or drugs to get sex or using sex to get gifts drugs, or other items of value). Chemsex was defined as the use of methamphetamine, GHB, cocaine, MDMA, ketamine, or synthetic cathninones within two hours before or during sex.

Participants reported if they ever received a prescription for formal PrEP from a healthcare provider, confidence in speaking with a healthcare provider about sexual health and PrEP, belief that formal PrEP is an effective HIV prevention tool, and ease of finding HIV antiretroviral medications for informal PrEP outside of formal healthcare settings and on geosocial dating/sex apps for GBM (e.g., Grindr). Finally, participants reported awareness of long-acting injectable PrEP and “on-demand” or 2:1:1 dosing for PrEP.

## Analysis

Measures were dichotomized to indicate endorsement of a characteristic or behavior versus no endorsement. Bivariate logistic regression models were constructed to predict informal PrEP use among GBM on measures of demographics and background; sexual behaviors; and formal PrEP experience, knowledge and attitudes. All analyses were conducted using IBM SPSS Statistics ver. 27.

## Results

There were 196 participants who were eligible and included in the present analyses. Mean age was 33.4 (SD 8.56; range 19–55). Participants identified as white (*N* = 125; 63.8%), African American/Black (*N* = 31; 15.8%), Hispanic (*N* = 26; 13.3%), and other race/ethnicity (*N* = 13; 6.6%). One participant refused to report their race/ethnicity. Participants resided in all regions of the US and represented 37 states and the District of Columbia.

Table 1 shows that of the total sample, 10.7% (*n* = 21) reported informal PrEP use. Compared to those who have not, men reporting informal PrEP more frequently reported past year sexually transmitted infections (OR: 4.983; 95% CI: 1.928–12.879), group sex (OR: 4.733; 95% CI: 1.856–12.074), public sex in a gay bathhouse or sauna (OR: 5.053; 95% CI: 1.856–13.752), and transactional sex (OR: 9.222; 95% CI: 2.984–28.501). In addition, men reporting informal PrEP use also more frequently reported ever receiving a prescription for formal PrEP (OR: 4.222; 95% CI:1.664–10.715 1–4), ease of finding diverted HIV antiretroviral medications on geosocial dating/sex apps for GBM (OR: 3.037; 95% CI: 1.193–7.728), and awareness of “on-demand” PrEP (OR: 3.278; 95% CI: 1.295–8.296).

**Table 1:** Bivariate logistic regression models predicting informal use of antiretroviral medication for HIV prevention (*N* = 196)

	Informal PrEP		No Informal PrEP		OR	95% CI
	*N* = 21	10.7%	*N* = 175	89.3%		
**Demographics and Background**						
Age 18–24	1	4.8%	28	16.0%	0.263	0.034, 2.036
Hispanic	3	14.3%	23	13.1%	1.101	0.301, 4.036
African American/Black	6	28.6%	25	14.3%	2.400	0.851, 6.771
White	12	57.1%	113	64.6%	0.732	0.292, 1.832
Other race/ethnicity	0	0.0%	13	7.4%	0.000	0.000, 0.000
College degree or higher	15	71.4%	127	72.6%	0.945	0.346, 2.577
Residency in large central metropolitan area	10	47.6%	78	44.6%	1.131	0.457, 2.800
Residency in state with positive LGBTQ + policy	10	47.6%	83	47.4%	1.008	0.407, 2.494
Syphilis, gonorrhea, or chlamydia diagnosis^a^	10	47.6%	27	15.4%	4.983***	1.928, 12.879
**Sexual Behaviors**						
Number of male sex partners^b^					1.012	0.970, 1.056
Chem sex^c^	5	23.8%	25	14.3%	1.875	0.631, 5.576
Group sex^a^	11	52.4%	33	18.9%	4.733***	1.856, 12.074
Public sex (e.g., sauna, bathouse)^a^	8	38.1%	19	10.9%	5.053**	1.856, 13.752
Transactional sex^a^	7	33.3%	9	5.1%	9.222***	2.984, 28.501
Sex with females^b^	7	33.3%	48	27.4%	1.323	0.503, 3.476
**PrEP Experience**, **Knowledge and Attitudes**						
Ever received PrEP prescription from healthcare provider	12	57.1%	42	24.0%	4.222*	1.664, 10.715
Confident to speak to a healthcare provider about PrEP	16	76.2%	135	77.1%	0.961	0.565, 1.632
PrEP is an effective HIV prevention tool	19	90.5%	133	76.0%	3.000	0.671, 13.415
Ease of finding informal PrEP outside of healthcare setting	11	52.4%	58	33.1%	2.219	0.891, 5.526
Ease of finding informal PrEP on Grindr or similar apps	13	61.9%	61	34.9%	3.037*	1.193, 7.728
Awareness of long-acting injectable PrEP	10	47.6%	50	28.6%	2.273	0.908, 5.686
Awareness of “on-demand” or “2:1:1” dosing for PrEP	10	47.6%	38	21.7%	3.278*	1.295, 8.296

*P < 0.05. **P < 0.01. *** *P* < 0.001

^a^Past year; ^b^Past 6 months; ^c^Past 90 days

## Discussion

This is the first apparent study to examine informal PrEP use among a national sample of GBM in the US. Findings suggest that men in the sample who reported informal PrEP use would appear to benefit from formal PrEP, considering their participation in sexual behaviors associated with increased likelihood of HIV transmission (e.g., group sex, public sex, transactional sex). This may be especially true if informal PrEP is taken in a way that does not cohere with recommended dosage guidelines, which affords incomplete protection. Moreover, knowledge, acceptability, and use of formal PrEP appears to be high. Thus, additional research is needed to better understand this phenomenon and motivations for informal PrEP use as well as rejection of or desisting from formal PrEP use, such as enacted stigma by healthcare providers, anticipated stigma on behalf of GBM, and barriers to insurance participation or meeting required follow-up visit requirements [7]. It should also be noted that studies show that medications used for informal PrEP are obtained from a range of sources, including false requests for HIV post-exposure prophylaxis in healthcare settings or dating/sex apps targeted toward GMB (e.g., Grindr) [4, 5, 11]. Data related to the full context of informal PrEP would be especially informative given recent efforts to examine how to best incorporate formal PrEP into existing HIV prevention programs and healthcare settings serving GBM [8], and ongoing legislative action in multiple US states which allow pharmacists to prescribe and dispense formal PrEP directly to patients [12].

Although uptake of formal PrEP among GBM is increasing, better communication targeted toward GBM is necessary. Individuals engaging in informal PrEP use may be at risk for HIV infection if they do not follow recommended dosing and medical monitoring [13]; informal PrEP use has the potential to contribute to antiretroviral medication resistance [13]; and adherence to dosage guidelines is necessary to maintain effectiveness [1]. Research suggests that GBM engaging in informal PrEP use are using a range of antiretroviral medications and dosage practices, resulting in at least some examples of GBM becoming HIV positive after engaging in informal PrEP use [4, 6]. Thus, every effort should be made to connect GBM with formal PrEP and associated medical monitoring.

One such avenue to do so is through social media where many existing campaigns are attempting to increase awareness, uptake, and adherence. A majority of men reporting informal PrEP use, and a sizable plurality of the overall sample, endorsed the ease of finding HIV antiretroviral medications for informal PrEP on Grindr or similar geosocial dating/sex apps which are targeted specifically to GBM. Although prior work documents diversion of HIV antiretroviral medications, including for PrEP, on Grindr and other apps [11], additional research shows the promise of using these apps to link GBM into formal PrEP care [14]. Continued efforts utilizing GBM-focused social media may serve to mitigate informal PrEP use, limit inaccurate PrEP information in these spaces, and increase formal PrEP uptake. Our finding that GBM who were aware of event-driven PrEP were more than two times as likely to engage in informal use may indicate that more nuanced, accurate public health messaging is required to ensure that on-demand PrEP is not used inappropriately or incorrectly.

This study has some limitations worth noting. The ability to generalize the findings to other GBM is limited by the eligibility requirements, an unrepresentative sample, and online recruitment strategy. All data are based on self-report, potentially leading to under-reporting of socially undesirable behaviors. Causality cannot be inferred given that the data are cross-sectional and retrospective. Finally, the survey did not inquire about motivations for informal PrEP, adherence, or potential consequences. Longitudinal research is needed to further elucidate informal PrEP practices.

PrEP is a revolutionary HIV prevention tool. Thus, every effort should be made to encourage formal PrEP uptake, address barriers to formal PrEP which may encourage informal use, and resolve any potential misinformation surrounding PrEP medication and dosing. Such actions would likely mitigate any potential negative consequences of informal use.

## Data Availability

Data are available by request.
